# Single‐Molecule Imaging of Endogenous RNA Using a Miniaturized and Circularly Permuted CRISPR‐Cas13 Degron‐Masking System CDegSR

**DOI:** 10.1002/advs.77533

**Published:** 2026-08-31

**Authors:** Shipeng Shao, Hongchen Zhang

**Affiliations:** ^1^ Center For Medical Epigenetics School of Basic Medical Sciences National Clinical Research Center for Child Health and Disorder Children's Hospital of Chongqing Medical University Chongqing Medical University Chongqing China; ^2^ Department of Psychiatry Key Laboratory of Major Brain Disease and Aging Research (Ministry of Education) The First Affiliated Hospital of Chongqing Medical University Chongqing China; ^3^ Integrative Science Center of Germplasm Creation in Western China (Chongqing) Science City MOE Key Laboratory of Freshwater Fish Reproduction and Development, School of Life Sciences Southwest University Chongqing China

**Keywords:** background rejection, CDegSR, circular permutation, CRISPR, degron, RNA tracking, single molecule imaging

## Abstract

Visualizing the spatiotemporal dynamics of endogenous, unmodified RNA within living cells is crucial for understanding gene regulation. Yet, existing techniques are often hindered by high background fluorescence and the steric hindrance posed by CRISPR effectors. To address this, we developed CDegSR (CRISPR‐mediated Degron‐based Signal Restoration), a compact, fluorogenic system for high‐contrast RNA imaging at single‐molecule resolution. By miniaturizing d*Psp*Cas13b to its essential recognition lobe and employing circular permutation, we repositioned the protein termini to enable target‐dependent masking of a potent degron. This approach promotes rapid proteasomal degradation of effectors not bound to targets while selectively stabilizing those attached to target RNA. Using CDegSR, we achieved single‐molecule tracking of endogenous *POLR2A* transcripts, revealing that nuclear crowding and the translation machinery are the main physical constraints on mRNA mobility. CDegSR provides a versatile, low‐perturbation toolkit for dissecting the transcriptomic lifecycle at single‐molecule resolution.

## Graphical abstract

CDegSR, a new tool for imaging RNA at single molecule level in living cells was developed by researchers. Background noise was eliminated by protein engineering and making it degrade when not bound to its target. This allowed individual RNA molecules to be clearly imaged and tracked.

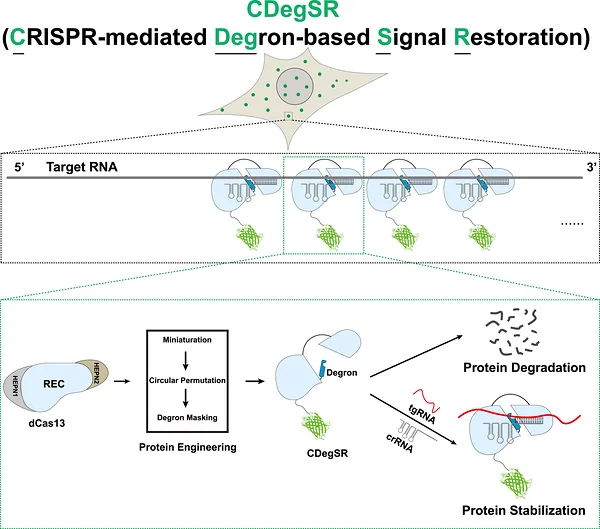

## 1 Introduction

RNA functions as the central intermediary in the flow of genetic information, governed by complex spatiotemporal regulations that ensure proper cellular function. Aberrations in RNA metabolism and localization are critically associated with a broad spectrum of pathologies, including malignancy and neurodegenerative disorders. Consequently, the capacity to visualize endogenous RNA dynamics in living cells is indispensable for decoding the complex life cycle of the transcriptome [1]. Although single‐molecule RNA fluorescent in situ hybridization (smFISH) offers exceptional sensitivity and spatial resolution, its requirement for cell fixation precludes the observation of real‐time molecular behaviors [2, 3].

To address this limitation, live‐cell imaging methodologies, including the MS2/PP7 systems [4, 5] and fluorogenic aptamers [6, 7, 8, 9, 10, 11], have been developed. However, these approaches typically require extensive genetic tagging, which may perturb RNA stability and function. CRISPR‐based technologies, such as RCas9 [12], dCas13 [13, 14], and Csm [15], offer a programmable alternative for targeting unmodified endogenous RNAs. Despite this advantage, these systems frequently exhibit elevated background fluorescence attributable to the presence of non‐target‐bound effectors. Additionally, the considerable molecular size of conventional Cas proteins imposes a significant steric hindrance on the target RNA and complicates delivery through size‐constrained vectors, such as adeno‐associated viruses. These challenges underscore the imperative for more compact, user‐friendly tools that minimizes perturbation while maintaining high sensitivity and low background signal.

Here, we present CDegSR (CRISPR‐mediated Degron‐based Signal Restoration), a compact and fluorogenic platform for high‐contrast RNA imaging with single‐molecule sensitivity. Truncating d*Psp*Cas13b to its minimal recognition lobe and utilizing circular permutation allowed us to reposition the protein termini, achieving target‐activated conditional masking of the degron [16, 17, 18, 19]. This approach ensures rapid degradation of unbound effectors, while selectively stabilizing RNA‐bound complexes, thereby substantially improving the signal‐to‐noise ratio. Using CDegSR, we successfully performed single‐molecule tracking of endogenous *POLR2A* transcripts, uncovering the effects of nuclear crowding and the translational machinery on mRNA mobility. This versatile, minimally perturbative toolkit provides a robust platform for investigating RNA dynamics at single‐molecule resolution.

## 2 Results

### 2.1 Engineering and Characterization of a Miniaturized dPspCas13b (miniCas13) for RNA Imaging

The demand for compact, efficient RNA imaging tools is driven by the need to visualize endogenous transcripts in living cells with minimal steric or functional perturbation. Smaller protein effectors are particularly desirable for their reduced steric hindrance on target RNAs (tgRNAs) and their suitability for delivery systems with limited cargo capacity. To this end, we aimed to engineer a miniaturized variant of the catalytically inactive *Prevotella sp*. Cas13b (d*Psp*Cas13b) [20], hereafter termed miniCas13, by truncating non‐essential domains while retaining high‐affinity RNA‐binding capability.

**FIGURE 1:**
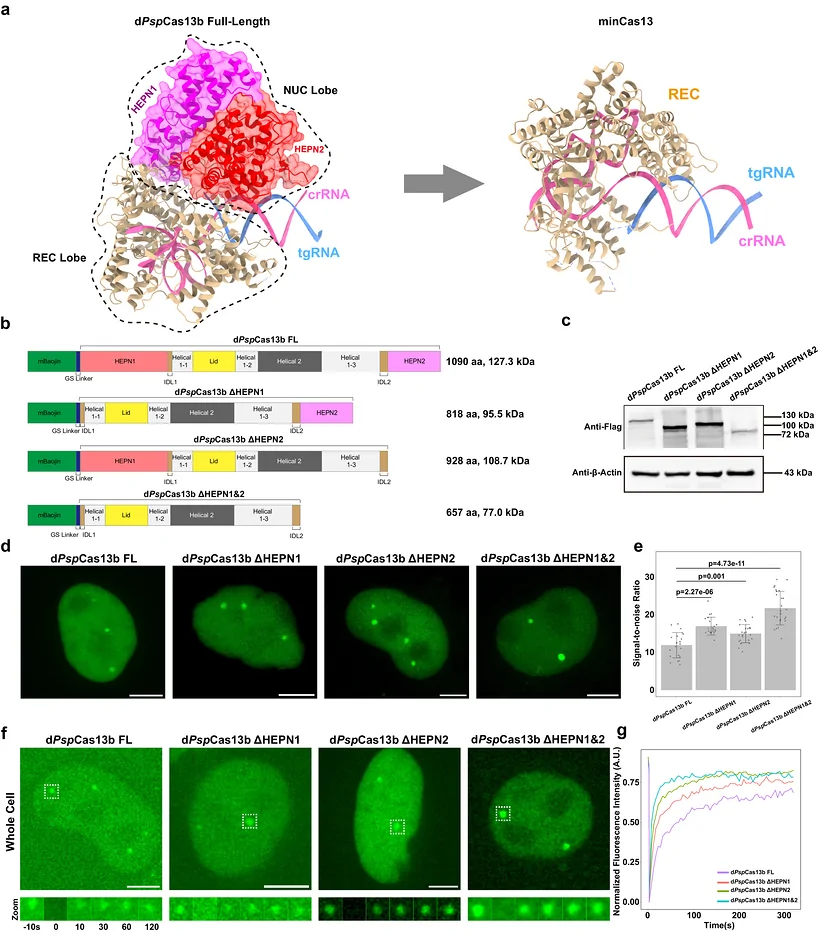
Engineering and Characterization of a Compact Cas13 Variant mini‐dCas13 for Enhanced RNA Imaging. (a) Structural modeling and minimization strategy. Three‐dimensional representations of the *Psp*Cas13b–crRNA–tgRNA ternary complex (left, based on PDB: 8WFA) and the engineered recognition (REC) lobe‐only variant (right). The HEPN1 (purple) and HEPN2 (red) domains within the nuclease (NUC) lobe are highlighted to illustrate the truncation regions targeted for structural miniaturization. (b) Domain architecture of d*Psp*Cas13b variants. Schematic diagrams illustrating the primary sequence organization of full‐length (FL) d*Psp*Cas13b and the derived truncation mutants: V1 (ΔHEPN1), V2 (ΔHEPN2), and V3 (ΔHEPN1&2, designated as miniCas13). Amino acid (aa) lengths and predicted molecular weights (kDa) are detailed for each construct. (c) Expression and protein integrity of d*Psp*Cas13b variants. Immunoblot analysis of HeLa cell lysates verifying the stable expression and molecular integrity of the mBaojin‐fused d*Psp*Cas13b variants. (d) Endogenous RNA labeling performance using d*Psp*Cas13b variants. Representative fluorescence images of HeLa cells demonstrating the localization of various d*Psp*Cas13b variants to endogenous *NEAT1* lncRNA within nuclear paraspeckles. Scale bars, 5 µm. (e) Quantification of imaging contrast of d*Psp*Cas13b variants. Statistical assessment of the signal‐to‐noise ratio (SNR) for *NEAT1* foci. Data are mean±SD. Significance was determined by one‐way ANOVA, followed by Dunn's multiple comparison test. P values were labeled up each bar. For each bar from left to right, n = 22, 21, 26, and 26 cells, respectively. (f, g) Dynamic stability and exchange kinetics of *NEAT1* labeled by d*Psp*Cas13b. Representative FRAP time‐lapse sequences (f) and normalized recovery curves (g) for *NEAT1* foci. Magnified insets track the fluorescence recovery of individual puncta. Scale bars, 5 µm.

Our engineering strategy was informed by the cryo‐electron microscopy (cryo‐EM) structure of the *Psp*Cas13b–crRNA–tgRNA ternary complex (PDB#8WFA), which reveals a bilobed architecture [21]. This structure consists of a recognition (REC) lobe containing the Helical‐1, Lid, and Helical‐2 domains and a nuclease (NUC) lobe primarily formed by the HEPN1 and HEPN2 domains (Figure 1a). The REC lobe anchors the crRNA direct repeat (DR), whereas the NUC lobe is responsible for ribonuclease activity, which is already inactivated in the catalytically dead d*Psp*Cas13b variant. Previous studies have demonstrated that deletion of the HEPN2 domain (ΔHEPN2) preserves RNA editing function [21]. Thus, we hypothesized that complete removal of the NUC lobe (ΔHEPN1 and ΔHEPN2) could further optimize the effector's size and enhance accessibility to the guide‐target RNA duplex.

Guided by these insights, we constructed three truncation variants (V1–V3) based on the d*Psp*Cas13b scaffold: V1 (ΔHEPN1), V2 (ΔHEPN2), and V3 (ΔHEPN1&2). Each variant was fused to the bright green fluorescent protein mBaojin [22] and a nuclear localization signal (NLS) (Figure 1b). Immunoblot analysis confirmed stable expression of all variants as full‐length fusion proteins in mammalian cells, with molecular weights consistent with their theoretical designs (Figure 1c). To evaluate the RNA‐labeling efficacy of the engineered variants, we targeted the long non‐coding RNA (lncRNA) *NEAT1*, a well‐established benchmark due to its distinct localization within nuclear paraspeckles [23]. Confocal microscopy of HeLa cells demonstrated that all three truncation variants (V1–V3) successfully localized to discrete nuclear foci (Figure 1d). Notably, variants lacking the HEPN2 domain (V2 and V3) exhibited significantly improved signal‐to‐noise ratios (SNR) relative to the full‐length (FL) d*Psp*Cas13b (Figure 1e). The dual‐deletion variant, miniCas13 (V3), emerged as the most potent scaffold, suggesting that the excision of the bulky NUC lobe reduces steric constraints and improves the kinetics of guide‐target duplex engagement. To rigorously verify that the observed fluorescent puncta corresponded to authentic *NEAT1* transcripts rather than nonspecific protein aggregates, we performed orthogonal validation via immunofluorescence staining for PSF (a core paraspeckle protein) and RNA FISH for *NEAT1* transcripts. The pronounced spatial colocalization between mBaojin‐labeled foci and PSF‐stained structures/RNA FISH labeled foci confirmed the specificity and fidelity of the miniCas13 system in detecting endogenous RNA targets within their native subcellular compartments (Figure S1).

To investigate the impact of NUC lobe truncation on the stability of the Cas13:crRNA:tgRNA ternary complex, we performed Fluorescence Recovery After Photobleaching (FRAP) on *NEAT1* foci (Figure 1f). Interestingly, variant V3 exhibited the most rapid recovery kinetics compared to V1, V2, and full‐length d*Psp*Cas13b (V3> V2 = V1> FL) (Figure 1g and Figure S2). This accelerated exchange implies that deletion of the NUC lobe moderately destabilizes the ternary complex, a characteristic that paradoxically benefits imaging applications. Enhanced turnover facilitates continuous replacement of photobleached fluorophores at target sites, thereby sustaining signal persistence during prolonged imaging sessions.

Collectively, these findings demonstrate that the HEPN domains are dispensable for programmable RNA targeting and imaging. By reducing the effector to its minimal functional core (recognition (REC) lobe), we developed a streamlined scaffold exhibiting increased conformational flexibility and superior imaging performance. Given its compact size, compatibility with viral delivery, and optimized binding dynamics, we selected miniCas13 (V3) as the foundational scaffold for the development of our subsequent interaction‐dependent degradation systems.

### 2.2 Evaluating the Efficacy of C‐Terminal Degron Masking in miniCas13

Following the successful development of a streamlined miniCas13 scaffold, we initially explored whether the addition of a C‐terminal degradation signal could induce a fluorogenic response in the effector protein (Figure S3a). We employed an RRRG motif (Degron or Deg), an arginine‐rich degron known to facilitate rapid protein degradation via recruitment of the ubiquitin‐proteasome system [24]. We hypothesized that binding to the target RNA might sterically hinder this degron, thereby stabilizing the effector specifically upon formation of the ribonucleoprotein (RNP) complex.

Preliminary validation demonstrated the efficacy of the C‐terminal Degron fusion. Transfected HeLa cells exhibited negligible whole‐cell fluorescence in the absence of target RNA, indicating efficient and constitutive degradation of the unbound mBaojin‐miniCas13‐Deg fusion protein (Figure S3b,c). To evaluate target‐dependent stabilization, we designed an orthogonal reporter system targeting the mScarlet3‐H [25] sequence, which is absent from the native human transcriptome (Figure S3b). Unexpectedly, co‐expression of the cognate crRNA and target RNA failed to restore the fluorescent signal (Figure S3c). Quantitative confocal imaging revealed no significant increase in protein accumulation upon target engagement, suggesting that the degron remained functionally active despite RNA binding (Figure S3d). Structural analysis (PDB#8WFA) revealed that the native d*Psp*Cas13b C‐terminus is spatially distant from the RNA‐binding cleft, thereby precluding target‐dependent masking of the degron. As a result, the Degron remains accessible to E3 ubiquitin ligases even when bound to RNA. These findings imply that C‐terminal fusion alone is insufficient to achieve target‐dependent stabilization, necessitating a topological rearrangement to position the degron near the RNA‐binding interface.

### 2.3 Circular Permutation of miniCas13 to Enable Proximity‐Dependent Degron Masking

**FIGURE 2:**
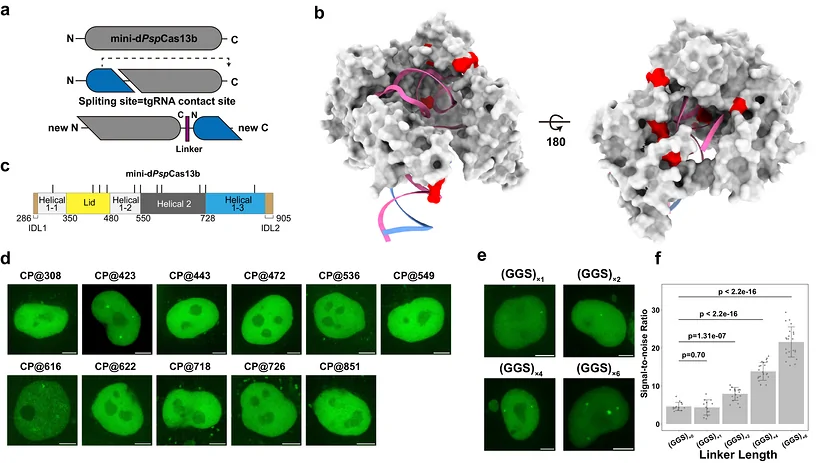
Topological Rearrangement and Optimization of the miniCas13 Scaffold via Circular Permutation. (a) Schematic workflow illustrating the design strategy for circular permutation (CP) of miniCas13, involving site‐specific internal cleavage and covalent fusion of the native N‐ and C‐termini via a flexible linker to redistribute miniCas13's topological orientation. (b, c) Spatial distribution (b) and linear sequence mapping (c) of 11 candidate cleavage sites within the miniCas13 structure, strategically selected based on their proximity to the target RNA‐binding interface and localization within flexible loop regions. (d) Representative fluorescence images demonstrating the labeling efficiency of endogenous *NEAT1* lncRNA using various miniCas13‐CP candidates. Scale bars, 5 µm. (e, f) Linker length optimization between the original termini using varying (GGS)_×n_ sequences. Quantitative analysis of the signal‐to‐noise ratio (SNR) for *NEAT1* foci (f) identifies a (GGS)_×6_ configuration as the most robust construct for high‐contrast intracellular RNA visualization. Scale bars, 5 µm. Data are mean±SD. Significance was determined by one‐way ANOVA, followed by Dunn's multiple comparison test. P values were labeled up each bar. For each bar from left to right, n = 14, 13, 19, 23, and 22 cells, respectively.

To circumvent the spatial constraints imposed by the native d*Psp*Cas13b termini, we hypothesized that relocating the degron to a site proximal to the RNA‐binding interface would allow the target RNA to hinder E3 ligase access sterically. Achieving this required a topological rearrangement of the miniCas13 scaffold via circular permutation (CP). CP can generate new proteins through covalently linking the original N‐ and C‐termini via a flexible linker and generating new termini by cleaving the protein at an alternative, structurally informed locus [26] (Figure 2a). By reconfiguring the connectivity of secondary structural elements while preserving the overall tertiary conformation, CP can expose internal loops to the solvent, thereby providing new attachment points for regulatory domains without compromising the effector's core functionality.

Inspired by prior successful applications of circular permutation in modulating the activities of other CRISPR effectors, including Cas9 [27] and CasRx [28], we implemented this strategy on our engineered miniCas13 scaffold. We established two primary criteria for selecting permutation sites: (1) proximity to residues that directly interface with the target RNA, and (2) localization within disordered or flexible loop regions to minimize disruption of the protein's core structural integrity. Utilizing the ternary complex structure of d*Psp*Cas13b as a reference, we initially identified 11 candidate sites for experimental validation. In these variants, the original termini were joined by a flexible glycine‐serine linker, and new termini were engineered adjacent to RNA‐contacting regions (Figure 2b,c).

We evaluated the RNA‐labeling functionality of the 11 CP variants in HeLa cells targeting endogenous *NEAT1* transcripts. While the majority of the permutations resulted in a loss of RNA‐binding activity, two variants—split at residues 423 and 616—retained the ability to form distinct nuclear foci. Among these, the variant permuted at position 423 (CP423) exhibited superior labeling robustness and was selected as the foundational scaffold for further optimization (Figure 2d).

We reasoned that the efficiency of the CP scaffold might be sensitive to the length and flexibility of the linker bridging the original N‐ and C‐termini. To test this, we engineered a series of CP423 variants with varying lengths of the glycine‐glycine‐serine (GGS)_×n_ linker. Quantitative fluorescence imaging of *NEAT1* foci revealed that a (GGS)_×6_ linker provided the optimal balance of structural stability and conformational flexibility, yielding the highest SNR (Figure 2e,f). This optimized variant, designated as miniCas13‐CP423, maintains high‐affinity RNA binding and full compatibility with standard crRNAs, providing a strategically positioned platform for the subsequent insertion of the Degron tag.

### 2.4 Development of CDegSR for Background Rejection

**FIGURE 3:**
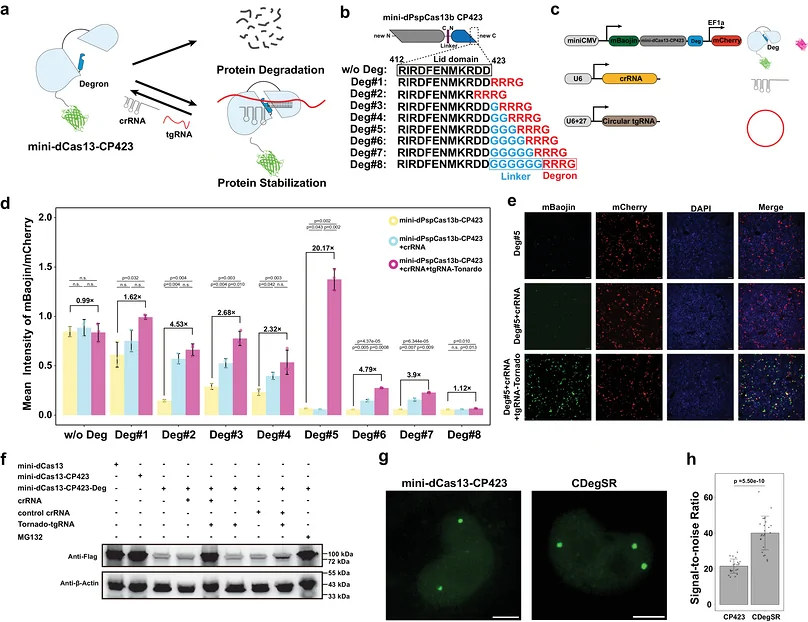
Engineering and Characterization of the Target‐Responsive, Conditionally Stable CDegSR Platform. (a) Schematic of the interaction‐dependent stabilization mechanism. The engineered miniCas13‐CP423 scaffold is fused to a C‐terminal RRRG degron, which recruits the ubiquitin‐proteasome machinery for rapid protein turnover in the unbound state. Programmed engagement with a cognate target RNA (tgRNA) sterically masks the degron, rescuing the ribonucleoprotein complex from degradation and restoring fluorescence. (b) Structural topology and degron integration sites. The diagram delineates the precise circular‐permutation site within the miniCas13‐CP423 scaffold and identifies the specific C‐terminal attachment points used to integrate the degron across various candidate constructs. (c) Overview of the stabilization assay components, including the miniCas13‐CP423‐Deg fusion protein, its directing crRNA, and an orthogonal Tornado‐structured circular tgRNA used as a stable, non‐native benchmark. (d) Quantitative assessment of variant stability. Data represent the normalized fluorescence intensities of multiple mBaojin‐miniCas13‐CP423‐Deg variants. Stability was evaluated under triple conditions: in the presence or absence of the circular target RNA and its guiding crRNA. Data are mean±SD from n = 3 independent field of views. Significance was determined by one‐way ANOVA, followed by Dunn's multiple comparison test. P values were labeled up each bar. (e) Representative fluorescence images of HEK293FT cells transfected with the optimized Degron #5 fusion constructs. Fluorescence signals were selectively detected only in cells co‐expressing both the Tornado‐structured target RNA and the complementary crRNA, demonstrating high specificity. Scale bars, 100 µm. (f) Immunoblotting analysis of protein levels. Western blot assays using anti‐Flag antibodies confirm the differential expression and stability of the Flag‐tagged Degron fusion variants across the tested conditions. (g, h) Enhanced imaging of endogenous transcripts. Representative fluorescence images of HeLa cells targeting *NEAT1* lncRNA (g) demonstrate that the CDegSR system provides significantly lower background noise and superior signal‐to‐noise ratios (SNR) compared to the constitutively fluorescent miniCas13‐CP423 (h). Scale bars, 5 µm. Data represent mean±SD. Significance was determined by unpaired student's t‐test. n = 22 for CP423 and n = 24 for CDegSR.

We then used the newly created miniCas13‐CP423 for protein degradation. To convert the constitutively fluorescent miniCas13‐CP423 protein into a fluorogenic one, we sought to couple its stability directly to its target‐binding state. We hypothesized that by rendering the unbound effector susceptible to rapid proteasomal turnover, fluorescence would be selectively restricted to the RNA‐bound fraction. To achieve this, we fused a potent RRRG degron to the C‐terminus of the miniCas13‐CP423 (Figure 3a).

We first investigated whether direct C‐terminal fusion of the RRRG motif to miniCas13‐CP423 could confer protein instability (Figure 3b,c). Upon expressing an mBaojin‐miniCas13‐CP423‐RRRG fusion in HEK293FT cells, we observed that the fluorescence intensity remained high and comparable to non‐degron controls, regardless of the presence of crRNA or Tornado [29]‐tgRNA (Figure 3d and Figure S4a). This suggested that the RRRG motif might be sequestered or neutralized by the local protein environment. We intended to eliminate potential charge neutralization of the arginine‐rich degron. Initial attempts to restore degradation by removing the distal aspartic acid (DD) residues yielded no improvement (Figure 3b–e). We reasoned that the lack of degradation might result from reduced accessibility of the RRRG motif to the proteasomal machinery. To increase the exposure of the degron, we inserted flexible glycine (G) linkers of varying lengths (1 to 6 amino acids) between the protein and the RRRG tag, creating a series of Deg variants (Figure 3b). Systematic evaluation revealed a linker‐length‐dependent decrease in basal fluorescence. As the linker extended from variant 3 to 8, fluorescence dropped by 30% to 95% in the absence of target RNA (Figure 3d and Figure S4). These results confirm that a flexible spacer is essential for efficient degron‐mediated turnover.

We next evaluated whether this degradation could be reversed upon target binding. While the expression of crRNA alone failed to stabilize the Deg variants, the addition of both crRNA and a circular tgRNA successfully rescued protein stability (Figure 3d–f and Figure S4). This is consistent with our design placing the circular permutation site near the tgRNA interface rather than the crRNA binding channel. Among the tested candidates, Deg variant 5 (containing a ‘GGG’ linker) emerged as the optimal configuration, demonstrating a twenty‐fold fluorescence induction upon target recognition. This variant, which we formally designated ‘CDegSR’, demonstrated consistent performance across diverse molecular reporters, including various fluorescent proteins and self‐labeling enzyme tags such as HaloTag and SNAP‐tag (Figure S5). Furthermore, the target‐dependent stabilization mechanism remained highly efficient across multiple cell lines, including HeLa, MDA‐MB‐231, and U2OS (Figure S6). To confirm that the background depletion of unbound CDegSR is indeed mediated by proteasomal degradation, we treated cells with the proteasome inhibitor MG132 and monitored protein expression levels via Western blotting. Notably, upon MG132 treatment, CDegSR remained highly stable even in the absence of the crRNA and target RNA (Figure 3f). This rescue effect demonstrates that the background suppression of our platform is strictly driven by the ubiquitin‐proteasome machinery. Finally, we applied the CDegSR system to visualize endogenous *NEAT1* RNA (Figure 3g). Compared to the parental miniCas13‐CP423, the CDegSR system yielded significantly lower background noise and a superior SNR, establishing it as a highly sensitive tool for live‐cell RNA imaging (Figure 3h). Furthermore, the colocalization of CDegSR and FISH targeting *NEAT1* in various cell lines demonstrate the robust target specificity, high labeling efficiency, and broad applicability of CDegSR for endogenous RNA imaging (Figure S7).

### 2.5 Single Molecule Imaging of Endogenous RNA Using CDegSR Tiling

**FIGURE 4:**
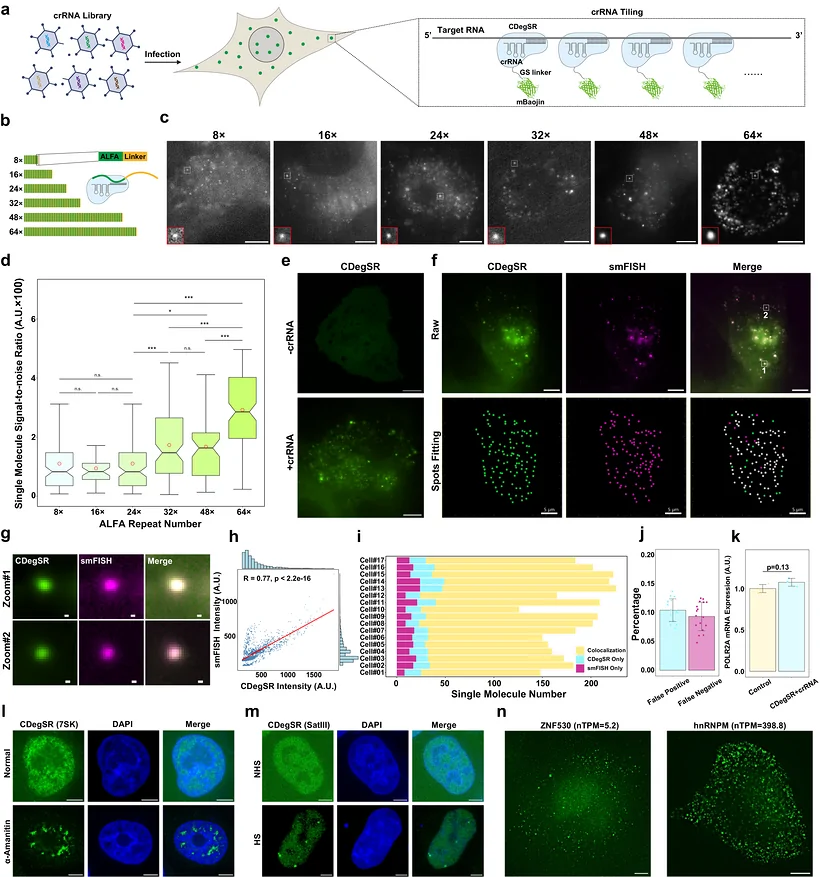
Single‐molecule Visualization and Orthogonal Validation of Endogenous Transcripts via CDegSR Tiling. (a) Schematic of the tiled RNA labeling strategy. High‐sensitivity, single‐molecule resolution is achieved by recruiting multiplexed RNP complexes to a single transcript. Intracellularly assembled CDegSR effectors, each programmed with unique crRNA spacers, simultaneously dock onto target sequences via base‐pair complementarity, facilitating synergistic signal amplification for the detection of individual molecules. (b–d) Synthetic reporter calibration. Schematic (b) illustrates the modular architecture of ALFA‐tag arrays, featuring repetitive peptide‐epitope sequences interleaved with flexible RNA linkers to minimize steric hindrance between recruited effectors. High‐resolution images (c) display discrete ALFA‐repeat transcripts labeled by the CDegSR system, with magnified insets highlighting individual, diffraction‐limited mRNA molecules. Quantitative SNR analysis (d) reveals a progressive improvement in detection clarity as repeat valency increases. Scale bars: 5 µm. Significance was determined by one‐way ANOVA, followed by Tukey HSD's multiple comparison test. For each box from left to right, n = 110, 39, 110, 106, 59, and 122 molecules, respectively. ***P_24×‐32×_ = 6.67 × 10^−4^; *P_24×‐48×_ = 2.21 × 10^−2^; ***P_32×‐64×_ = 3.60 × 10^−4^; ***P_48×‐64×_ = 4.82 × 10^−4^; ***P_24×‐64×_ = 3.60 × 10^−4^. (e) Targeting specificity for endogenous *POLR2A*. Representative images demonstrate the specific labeling of native *POLR2A* transcripts in HeLa cells with or without crRNA. Scale bars: 5 µm. (f, g) Orthogonal smFISH validation. Dual‐color co‐registration between CDegSR signals (mBaojin) and smFISH probes (AF647) targeting *POLR2A*. Merged images (f) and zoomed‐in (g) illustrate the high spatial overlap between the two channels. The single molecule signals were fitted as spots of both channels, and colocalized molecules were rendered as white. Scale bars: 5 µm (f) and 100 nm (g). (h) Spatial correlation analysis of CDegSR and smFISH signals yields a Pearson correlation coefficient of r = 0.77. (i) Quantification of the colocalization of CDegSR and smFISH signal in multiple cells. (j) The false‐positive and false‐negative percentage of CDegSR for mRNA labeling. (k) Relative expression level of *POLR2A* in control and CDegSR transfected cells. Actin was used as internal control. Data represent mean±SD. Significance was determined by unpaired student's t‐test. n = 3 for each group. (l) 7SK RNA lableing using CDegSR under normal and α‐Amanitin inhibition conditions. Scale bars: 5 µm. (m) SatIII RNA lableing using CDegSR under non‐heat shock (NHS) and heat shock (HS) conditions. Scale bars: 5 µm. (n) *ZNF530* and *hnRNPM* RNA labeling using CDegSR. nTPM, normalized Transcripts Per Million. nTPM data were obtained from the Human Protein Atlas. Scale bars: 5 µm.

While our initial results demonstrated that the CDegSR system could label *NEAT1* RNA with high SNR, we sought to determine if this platform could be scaled to achieve single‐molecule resolution for endogenous transcripts. Visualizing individual RNA molecules in living cells requires the recruitment of multiple fluorescent effectors to a single target to overcome background fluorescence and the limits of diffraction‐limited detection (Figure 4a). To achieve this, we hypothesized that the multimerization of the ALFA‐tag [30], a compact and high‐affinity peptide epitope, would provide the necessary stoichiometric amplification of fluorescence signal (Figure 4b). To construct these repetitive arrays, we used isocaudomer assembly (Figure S8). To ensure structural flexibility and prevent steric hindrance between recruited effectors, a flexible RNA linker was inserted between each ALFA repeat. We engineered a library of reporter constructs containing varying repeat valencies, specifically 8×, 16×, 24×, 32×, 48×, and 64× ALFA tags (Figure 4b).

Evaluation of these constructs revealed a direct, non‐linear correlation between repeat number and fluorescence signal (Figure 4c). While the CDegSR effector, guided by crRNAs targeting these tandem arrays, showed detectable fluorescence at lower valencies, a threshold of at least 8× repeats was necessary to produce high‐contrast puncta that were distinguishable from the background. As valency increased, the SNR improved progressively (Figure 4d). We identified the 24× ALFA array as the optimal balance for single‐molecule detection, which provided sufficient brightness to resolve individual transcripts clearly while minimizing the genetic burden and the potential for mRNA mislocalization associated with excessively large crRNA targeting (e.g., 64×).

Transitioning from synthetic reporters to endogenous targets, we focused on labeling RPB1 (*POLR2A*), the largest subunit of the transcription machinery. To facilitate robust labeling, we designed a library of 24 distinct crRNAs tiling the *POLR2A* sequence using the *cas13target* computational suite to ensure high on‐target efficiency and minimal off‐target activity within the human transcriptome [31]. To overcome the challenges of delivering multiple crRNAs into a single cell, we utilized high‐titer lentiviral libraries. This approach enabled simultaneous delivery of a diverse crRNA population, ensuring that each endogenous *POLR2A* molecule could be “coated” with sufficient CDegSR effectors. We established a stable cell line expressing the CDegSR effector and subsequently transduced these cells with the *POLR2A*‐targeting lentiviral library. In the absence of targeting crRNAs, the CDegSR signal remained negligible, characterized by weak, diffuse fluorescence throughout the cell. Conversely, cells transduced with the *POLR2A* crRNA library exhibited bright, discrete fluorescent foci distributed within both the cytoplasm and the nucleus, consistent with the known localization patterns of *POLR2A* mRNA (Figure 4e).

To rigorously confirm that these fluorescent puncta represented authentic *POLR2A* transcripts rather than non‐specific protein aggregates or off‐target binding, we performed orthogonal validation using single‐molecule fluorescence in situ hybridization (smFISH). Fixed cells were hybridized with Alexa Fluor 647 (AF647)‐conjugated antisense oligonucleotide probes specifically targeting the *POLR2A* sequence (Figure 4f,g). Dual‐color imaging demonstrated a high degree of spatial co‐registration between the CDegSR puncta and the smFISH signals. Quantitative colocalization analysis revealed a strong Pearson correlation coefficient (r = 0.77) between the two channels (Figure 4h). To further quantify the error rates of CDegSR labeled RNA, we counted the puncta number labeled by CDegSR only, smFISH only, and both simultaneously (Figure 4i). Notably, both the false‐positive and false‐negative rates were below 10% (Figure 4j). Additionally, targeting *POLR2A using* CDegSR does not significantly alter the abundance or turnover of the transcript (Figure 4k). These findings collectively demonstrate that the CDegSR system, combined with a tiled crRNA strategy, enables highly specific, sensitive, and single‐molecule resolution mapping of endogenous RNAs.

To establish the versatility of CDegSR, we expanded our imaging targets to diverse genomic transcripts. We first labeled the non‐coding RNA 7SK, a key regulator of RNA Pol II transcription elongation. While 7SK was diffusely distributed within the nucleoplasm under basal conditions, α‐Amanitin‐mediated inhibition of RNA Pol II induced its condensation into large clusters (Figure 4l), validating prior reports [32]. We next targeted SatIII, an ncRNA typically expressed at basal levels with no discernible spatial organization. Under heat shock, HSF1‐driven transcriptional bursts of SatIII led to the rapid assembly of robust transcription hubs [33] (Figure 4m). To test the detection sensitivity of CDegSR across varying expression levels, we targeted mRNAs ranging from the low‐abundance *ZNF530* (nTPM = 5.2) to the highly expressed *hnRNPM* (nTPM = 398.8). Both targets were successfully resolved following crRNA tiling (Figure 4n). Collectively, these findings highlight CDegSR as a robust and generalizable tool for imaging a diverse spectrum of RNAs, regardless of their type (ncRNA vs. mRNA) or expression abundance.

### 2.6 Tracking Endogenous mRNA Dynamics Using cDegSR System

**FIGURE 5:**
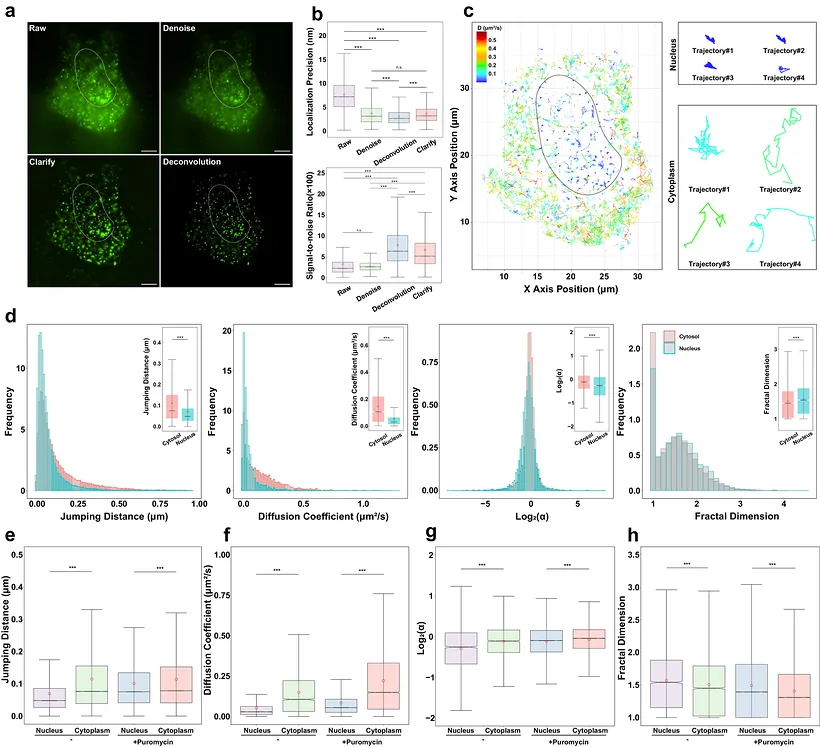
Real‐time Spatiotemporal Tracking and Biophysical Characterization of Endogenous mRNA Dynamics. (a) Representative fluorescence imaging of *POLR2A* mRNA. Raw image and post‐processed images of individual transcripts in living HeLa cells were shown. White dashed lines delineate the nuclear envelopes. Scale bars, 5 µm. (b) Quantitative assessment of image processing performance. Comparison of localization precision and signal‐to‐noise ratio (SNR) across distinct computational frameworks to validate the fidelity of single‐molecule tracking. Significance was determined by one‐way ANOVA, followed by Tukey HSD's multiple comparison test. For each box from left to right, n = 44641, 62659, 105485, and 104693 molecules, respectively. Localization precision: ***P_Raw‐Denoised_<2.2×10^−16^; *P_Raw‐Decon_<2.2×10^−16^; ***P_Raw‐Clarified_<2.2×10^−16^; ***P_Denoised‐Decon_<2.2×10^−16^; ***P_DeconDecon_<2.2×10^−16^. SNR: ***P_Denoised‐Clarified_<2.2×10^−16^; *P_Raw‐Decon_<2.2×10^−16^; ***P_Raw‐Clarified_ = 2.75×10^−14^; ***P_Denoised‐Decon_<2.2×10^−16^; ***P_DeconDecon_<2.2 ×10^−16^. (c) Spatiotemporal mapping of mRNA motility. Typical trajectories of individual *POLR2A* mRNA molecules in the nucleoplasm and cytoplasm are visualized. Trajectories are color‐coded by the magnitude of their diffusion coefficients, with magnified insets providing a detailed view of localized diffusion behavior. (d) Comprehensive statistical analysis of mRNA diffusion kinetics. Distribution of four key biophysical parameters were compared between the nucleus and cytoplasm. Significance was determined by two sample Wilcoxon rank sum test. For each box from left to right, n = 3450, 3039 molecules for cytoplasm and nucleus. ***P<2.2×10^−16^ for all group. (e–h) Impact of translational state on mRNA dynamics. Comparative analysis of jumping distance (e), diffusion coefficient (f), scaling factor (g), and fractal dimension (h) under control conditions versus translation inhibition via puromycin treatment. Significance was determined by two sample Wilcoxon rank sum test. For each box from left to right, n = 3062, 3456, 43239, and 18380 molecules, respectively. ***P<2.2×10^−16^ for all group.

To characterize the spatiotemporal dynamics of endogenous *POLR2A* mRNA with high precision, we leveraged the CDegSR system for reliable single‐molecule tracking (SMT) within the complex environment of living cells. Recognizing that SMT performance requires an optimal SNR and high localization accuracy, we benchmarked three computational frameworks, Denoise.ai, Clarify.ai, and iterative Deconvolution to maximize signal integrity. While Denoise.ai refined localization precision from 7 nm to 4 nm by mitigating shot noise, and Clarify.ai effectively reduced out‐of‐focus blur, Deconvolution yielded the most substantial improvements in image sharpness and spatial resolution (Figure 5a,b). Given its superior contrast for individual *POLR2A* puncta, we adopted deconvolution as the standard pipeline for all subsequent dynamic analysis.

Long‐term, high‐frequency tracking revealed striking differences in kinetic profiles based on subcellular compartmentalization (Figure 5c). *POLR2A* molecules in the cytoplasm exhibited significantly higher diffusion coefficients and larger jumping distances compared to those in the nucleus. To characterize the nature of this motion, we calculated the scaling factor α and the fractal dimension of the trajectories (Figure 5d). Nuclear RNA showed a lower α value, characteristic of sub‐diffusive or constrained motion, suggesting that the dense nuclear architecture comprising chromatin territories, nuclear bodies, and proteins imposes physical constraints that restrict transcript mobility. In contrast, cytoplasmic RNA moved more freely, reflecting a less crowded environment.

Finally, we investigated whether the translational machinery influences the diffusive behavior of *POLR2A* mRNA. Following treatment with puromycin, which is an amino‐nucleoside antibiotic that causes premature chain termination and ribosome dissociation, we observed a global increase in mRNA mobility in both the nucleus and the cytoplasm. Notably, the diffusion coefficient of cytoplasmic *POLR2A* increased significantly upon translation inhibition (Figure 5e), suggesting that the stoichiometric load of the polyribosome complex acts as a “molecular anchor” that confines transcript movement. The release of these bulky complexes facilitates more rapid mRNA diffusion through the cytosol. Interestingly, the moderate increase in nuclear mRNA mobility following puromycin treatment may imply a potential feedback mechanism that contributes to transcript confinement before cytoplasmic export, a mechanism that remains to be further explored (Figure 5f–h). Collectively, these data demonstrate that CDegSR is a powerful tool for dissecting the biophysical and functional constraints that govern the life cycle of endogenous RNA.

## 3 Discussion

In this study, we developed CDegSR, a fluorogenic CRISPR‐Cas13 platform that achieves high‐contrast, single‐molecule imaging of unmodified endogenous RNA in living cells. By integrating protein miniaturization, structural circular permutation, and a target‐dependent degron‐masking mechanism, the CDegSR system effectively overcomes the long‐standing challenge of background fluorescence from unbound CRISPR effectors. This allowed us to resolve the complex spatiotemporal dynamics of individual transcripts, providing new biophysical insights into the compartmentalized movement of RNA and the regulatory role of the translational machinery.

A primary innovation of this work is the systematic engineering of the *Psp*Cas13b scaffold. While traditional dCas13 systems utilize bulky, full‐length protein that may sterically hinder native RNA interactions, our miniCas13 variant, which lacks the non‐essential NUC lobe, optimizes the genetic payload for delivery vehicles like AAV. Interestingly, our FRAP analysis revealed that this truncation increases turnover kinetics. While lower complex stability might seem disadvantageous, it is paradoxically beneficial for long‐term imaging: faster exchange replenishes the fluorescence signal at target sites, effectively counteracting photobleaching.

The transition from a constitutive to a fluorogenic “switch” necessitated more than simple miniaturization. We identified a fundamental spatial mismatch where the native protein termini were too distal from the RNA‐binding cleft to achieve effective masking. Through structural circular permutation (CP423), we repositioned the degron‐attachment point proximal to the guide‐target RNA duplex. This topological rearrangement ensures that the Degron tag is sterically occluded and thus stabilized only upon mature RNP formation. This design effectively silences the “searching” pool of effectors, yielding superior signal‐to‐noise ratios.

Applying the CDegSR system to endogenous *POLR2A* transcripts allowed us to dissect the biophysical properties of the cellular environment with single‐molecule precision. Our findings that nuclear *POLR2A* mRNA exhibits significantly slower, sub‐diffusive motion compared to its cytoplasmic counterparts highlight the extreme molecular crowding within the nucleus. The dense network of chromatin and nuclear bodies acts as a physical restrict to the fractal dimension of RNA trajectories. Furthermore, our experiments with puromycin provide compelling evidence that the translational machinery serves as a major determinant of mRNA kinetics in the cytoplasm. The significant increase in diffusion coefficients upon ribosome dissociation suggests that mRNAs are not merely “free‐floating” but are instead heavily “anchored” by the massive stoichiometric load of the polyribosome complex.

The versatility of the CDegSR opens several avenues for future research. Firstly, the development of orthogonal Cas13 degron‐fluorescent protein pairs could enable the simultaneous, multicolor tracking of different RNA species (e.g., a coding mRNA and a non‐coding lncRNA) within the same cell. Secondly, the system could be integrated with proximity labeling (e.g., BioID or APEX) to map the dynamic “interactome” of a specific RNA molecule, thereby reducing background by degrading unbound probes. Finally, the compact nature of the CDegSR scaffold makes it an ideal candidate for in vivo applications via AAV delivery in animal models, enabling a new method for imaging and tracking single‐molecule RNA in living animals.

While the mature dMCP system utilizes high‐affinity aptamers for multicolor imaging [17, 18, 19], it requires tedious genomic insertion of dense stem‐loops to visualize endogenous RNAs. In contrast, CDegSR enables programmable, single‐molecule imaging of completely unmodified endogenous transcripts, bypassing host genome editing. Target reprogramming requires only synthesizing a new crRNA pool. Furthermore, cooperative fluorophore recruitment via a tiled crRNA strategy effectively compensates for dCas13's lower single‐site affinity, matching dMCP's excellent signal‐to‐noise ratio while fully preserving native transcript biology. To directly compare the performance of CDegSR and the gold‐standard single‐molecule RNA imaging tool MS2, we generated a reporter construct containing the *POLR2A* coding sequence appended with a 24× MS2 cassette in its 3′ UTR. We then monitored live‐cell single mRNA motility in parallel using either our tiled CDegSR platform or the conventional tandem MCP (tdMCP‐GFP) system. Our comparative trajectory analysis demonstrates that the movement dynamics of the mRNA labeled by CDegSR are highly comparable to those tracked by the MS2 system, showing negligible biophysical variance (Figure S9). This pivotal control confirms that the macromolecular payload of tiled CDegSR effectors does not artifactually compromise or alter the endogenous properties of the target transcript.

Beyond traditional aptamer‐based degron fusion, the recently developed CtDeg system achieves conditional stabilization of d*Psp*Cas13b through a fundamentally different strategy [34]. While CtDeg relies on RNA engineering through embedding a Pepper aptamer into the crRNA to control the stability of tDeg‐fused dCas13, our CDegSR platform is driven by intensive protein engineering. By combining d*Psp*Cas13b minimization with circular permutation, CDegSR achieves target‐dependent degron masking via intrinsic conformational changes, completely independent of specific aptamer sequences (Table 1).

**TABLE 1:** Comparison of Cas13‐Based Live‐Cell RNA Imaging Platforms.

Parameter / Feature	Conventional dCas13‐FP	CtDeg	CDegSR (This Work)
Effector Core Scaffold	Full‐length dCas13	Full‐length dCas13	Minimized miniCas13 (REC lobe core only)
CrRNA	Native	Engineered	Native
Effector Core Molecular Weight	∼130–150 kDa (bulky)	∼130–150 kDa (bulky)	∼70 kDa (ultra‐compact, half‐size)
Background Suppression Strategy	None (unbound effectors diffuse freely)	RNA Engineering: Embedding Pepper aptamer into crRNA scaffold	Protein Engineering: Circular permutation + intrinsic conformational degron‐masking
Aptamer/Ligand Dependency	None	Strictly requires specific Tat‐Pepper aptamer interaction	Completely independent of external aptamer sequences
Target RNA Modification	None	Requires complex guide/aptamer design optimization	None
Steric Hindrance to Cargo	High	High	Minimal (safeguards native mobility and transcript kinetics)
Signal‐to‐Noise Ratio (SNR)	Low to Moderate (high fluorescence background)	High (tDeg‐mediated background clearance)	High (cooperative tiled recruitment + efficient degron clearance)
Achievable Resolution	Limited to high‐abundance RNAs or large aggregates, repeat containing mRNA for single molecule imaging	Restricted to dense RNA clusters/aggregates (e.g., *NEAT1*, viral RNAs), did not show single molecule imaging	True Single‐Molecule Resolution (resolves low‐abundance individual transcripts)
Reprogramming Flexibility	High (swapping gRNA spacer)	Moderate (requires managing aptamer‐crRNA structural folding)	High (effortlessly redirected via a simple crRNA pool)

## 4 Conclusions

Altogether, the CDegSR system represents a major advancement in the RNA imaging toolkit. By harnessing the principles of conditional protein stability and structural engineering, we have created a high‐fidelity platform that not only visualizes where an RNA is but also reveals the biophysical environment it encounters throughout its life cycle. This technology will be instrumental in decoding the spatial logic of gene expression in both healthy physiology and disease states.

## 5 Experimental Section

### 5.1 Plasmid Construction

All plasmid constructs used in this study were detailed in Table S1. Target cDNA sequences were amplified from a HeLa S6 cDNA library generated by mRNA reverse transcription. Subsequently, all plasmids were generated by cloning these amplified fragments into their respective vectors using standard Gibson assembly.

To sequentially assemble tandem ALFA‐tag units, we employed a directional cloning strategy using the isocaudomers BglII and BamHI. Each unit comprised an ALFA‐tag sequence (SRLEEELRRRLTE) and a flanking linker to minimize steric hindrance between recruited CDeg effectors. The recipient vector containing the initial unit was linearized with BamHI and ligated with a BglII/BamHI‐digested insert containing the subsequent unit. Because BglII and BamHI generate compatible cohesive ends, their ligation forms a hybrid junction resistant to both enzymes. This directional assembly seamlessly preserves unique BglII and BamHI sites at the construct's extremities, enabling iterative cycles of digestion and ligation for continuous multimerization.

### 5.2 Cell Culture and Maintenance

HeLa S6, MDA‐MB‐231, HEK293FT, and U2OS cell lines were maintained in a humidified incubator at 37°C under 5% CO_2_. Culture media formulations varied by cell line: HeLa S6 and MDA‐MB‐231 cells were cultured in high‐glucose Dulbecco's Modified Eagle Medium (DMEM; Thermo Fisher Scientific, 11965092) supplemented with 10% fetal bovine serum (FBS; Excell Bio, FSD500) and 50 µg/mL each of penicillin and streptomycin (Thermo Fisher Scientific, 15140122). HEK293FT cells were propagated in high‐glucose DMEM (Thermo Fisher Scientific, cat. no. 11965092) containing 10% FBS (Excell Bio, FSD500), 0.1 mM non‐essential amino acids (NEAA; Thermo Fisher Scientific,11140050), 1% penicillin‐streptomycin (Thermo Fisher Scientific, 15140122), and 500 µg/mL G418 (Geneticin; MCE, HY‐17561 ). U2OS cells were grown in McCoy's 5A (Modified) medium (Thermo Fisher Scientific, 16600082) supplemented with 10% FBS and 1% penicillin‐streptomycin. All routine cell culture manipulations were performed using standard aseptic techniques. Cells were passaged upon reaching 80%–90% confluence using 0.25% trypsin‐EDTA. To prevent experimental artifacts, all cell lines were screened biweekly for mycoplasma contamination using a PCR‐based assay; only confirmed mycoplasma‐free cultures were used in subsequent experiments.

### 5.3 Transient Transfection

For transient transfection assays, cells were subcultured 24 h before the procedure and seeded into 35‐mm culture dishes at a density optimized to achieve 70%–80% confluence on the day of transfection. Transfections were performed using Lipofectamine 3000 reagent (Thermo Fisher Scientific, L3000015) in strict accordance with the manufacturer's instructions. Briefly, the appropriate concentrations of plasmid DNA and Lipofectamine 3000 were diluted independently in Opti‐MEM reduced‐serum medium (Thermo Fisher Scientific, 11058021). The diluted components were then mixed and incubated for 15–20 min at room temperature to allow for DNA‐lipid complex formation. Finally, the resulting complexes were added dropwise to the cells maintained in their respective complete growth media.

### 5.4 Live‐Cell Imaging

To visualize *NEAT1* and *POLR2A* mRNAs, 1 × 10^6^ HeLa S6 cells were electroporated with 10 µg of total plasmid DNA (CDegSR and the corresponding crRNA) in Opti‐MEM. Electroporation was performed using a Super Electroporator NEPA21 Type II with 2‐mm gap cuvettes. Subsequently, the cells were seeded into 35‐mm glass‐bottom dishes (20‐mm microwell, #1.5 cover glass; CellVis, cat. no. D35‐20‐1.5‐N) at a density of 4 × 10^5^ cells per well. After 48 h of incubation, the standard culture medium was replaced with a live‐cell imaging buffer consisting of phenol red‐free DMEM supplemented with 10% FBS and 1× penicillin‐streptomycin. Live‐cell imaging was conducted using a Nikon Ti2‐E inverted microscope equipped with a CrestOptics V3+DeepSIM spinning‐disk confocal system. Finally, all acquired images were processed and analyzed using ImageJ software (National Institutes of Health).

### 5.5 Immunofluorescence Staining

To perform PSF immunofluorescence staining, HeLa S6 cells were seeded at a density of 1.5 × 10^5^ cells per well onto 18‐mm‐diameter, #1.5‐thickness, collagen‐coated coverslips (Neuvitro) in a 12‐well culture plate. Following a 24‐hour incubation, the cells were co‐transfected with 2 µg of Neat1‐targeting CDegSR and the corresponding crRNA plasmids using 5 µL of Lipofectamine transfection reagent (Thermo Fisher Scientific). The cells were then cultured for 48 h to facilitate optimal protein and crRNA expression.

Post‐incubation, the cells were fixed using 4% paraformaldehyde (PFA; Electron Microscopy Sciences) in 1× phosphate‐buffered saline (PBS) for 10 min at room temperature. After washing three times with 1× PBS, permeabilization was achieved by incubating the cells with 0.5% (v/v) Triton X‐100 (Sigma) in 1× PBS for 10 min at room temperature, followed by an additional three washes in 1× PBS.

To minimize nonspecific binding, the samples were incubated in a blocking buffer containing 5% (w/v) bovine serum albumin (BSA; Jackson ImmunoResearch) in 1× PBS for 30 min. The cells were subsequently probed with a primary anti‐PSF antibody (Abcam, cat. no. EPR13359) diluted 1:200 in blocking buffer for 1 h at room temperature. After three 5‐minute washes in 1× PBS, the samples were incubated with an Alexa Fluor 647‐conjugated secondary antibody in blocking buffer for 1 h at room temperature. Following another three washes to remove unbound antibodies, the samples were post‐fixed with 4% PFA in 1× PBS for 10 min and washed a final three times (5 min each) in 1× PBS before mounting and imaging.

### 5.6 Fluorescence Recovery After Photobleaching (FRAP) Assay

FRAP experiments were performed using a Nikon Ti2‐E inverted microscope equipped with a 100× objective lens, a CrestOptics V3+DeepSIM spinning‐disk confocal system, and a GATACA Systems iLas2 targeted illumination module. To maintain optimal cell conditions, all imaging was conducted within an environmental control chamber.

Fluorescent signals corresponding to mBaojin‐labeled *NEAT1* foci were selected for analysis. For each analyzed cell, two circular regions of interest (ROIs), each encompassing a single fluorescent focus, were targeted. The imaging protocol commenced with the acquisition of 10 pre‐bleach frames captured at 200‐ms intervals to establish baseline fluorescence. Immediately following baseline acquisition, the selected ROIs were photobleached using the corresponding excitation laser at maximum intensity. To monitor fluorescence recovery, 100 post‐bleach frames were subsequently acquired at 1‐s intervals. During this recovery phase, the laser power for all channels was reduced to less than 20% transmission to minimize phototoxicity and incidental photobleaching.

For the quantitative analysis of recovery kinetics, raw fluorescence intensities from the bleached ROIs were first background‐subtracted. To account for acquisition‐related photobleaching and general background fluctuations, the data were normalized to an unbleached reference region within the same cell. Finally, the normalized intensities were scaled so that the average pre‐bleach intensity in each target ROI was set to 1.0, enabling precise evaluation of recovery dynamics.

### 5.7 Design and Preparation of the POLR2A crRNA Tiling Library

To achieve efficient endogenous labeling of the POLR2A transcript, we designed a tiling crRNA library specifically targeting its coding sequence (CDS) and 3' untranslated region (Table S2). This comprehensive targeting strategy was employed to maximize the available transcript surface area for optimal CDeg effector recruitment. Candidate crRNA spacers, ranging from 22 to 30 nucleotides in length, were generated and rigorously evaluated using the cas13target algorithm based on three primary filtering criteria: (1) a balanced GC content between 40% and 60%; (2) minimal off‐target potential; and (3) an optimal secondary structure folding energy to ensure robust target accessibility. Based on these parameters, a pool of 24 optimized spacer sequences was synthesized and subsequently cloned into the FUGW‐puro‐U6‐dPspCas13b‐crRNA lentiviral backbone utilizing a BsmBI‐mediated Golden Gate assembly protocol.

Lentiviral particles were generated employing a standard second‐generation packaging system. Briefly, HEK293FT cells were seeded into six‐well culture plates and cultured to reach approximately 90% confluence. Cells were then transiently co‐transfected using Polyethylenimine (branched, MW 25000, MCE, cat. no. HY‐W250110E) with a plasmid mixture containing 750 ng of the FUGW‐based transfer plasmid, 1.25 µg of the psPAX2 packaging plasmid, and 1.25 µg of the pMD2G envelope plasmid per well. Twenty‐four hours post‐transfection, the media were replaced with fresh complete growth media. At 48 h post‐transfection, the virus‐containing supernatants were harvested and clarified through a 0.45‐µm low‐protein‐binding filter to remove cellular debris. The purified viral preparations were either utilized immediately or aliquoted and stored at −80°C. For lentiviral transduction, HeLa S6 cells were infected by adding the filtered supernatant directly to the culture medium, supplemented with 8 µg/mL polybrene (MCE, cat. no. HY‐112735) to enhance transduction efficiency.

### 5.8 RNA Fluorescence in situ Hybridization (FISH)

To visualize endogenous mRNA targets, 30‐mm Petri dishes were first coated with 5 µg/mL poly‐L‐ornithine (Sigma‐Aldrich, cat. no. P4957) for 2 h at room temperature, followed by three thorough PBS washes. Cells were seeded onto the prepared dishes and cultured for 24 h. Subsequently, transient transfection with the RNA‐targeting constructs was performed as described above.

Forty‐eight hours post‐transfection, the cells were fixed with 4% paraformaldehyde (Electron Microscopy Sciences, cat. no. 1570) for 20 min at room temperature, and then permeabilized using 0.5% (v/v) Triton X‐100 (Sigma‐Aldrich, cat. no. X100) in 1× PBS for 10 min. Before hybridization, the samples were equilibrated for 5 min in a pre‐wash buffer consisting of 2× SSC (Thermo Fisher Scientific, cat. no. AM9765) and 30% (v/v) formamide (Thermo Fisher Scientific, cat. no. AM9344).

Primary hybridization was conducted overnight at 37°C in a humidified chamber. The custom POLR2A FISH probe pool (Sangon Biotech; Table S3) was applied at a total concentration of 200 nM (comprising approximately 40 individual oligonucleotides at 5 nM each). The hybridization buffer contained 2× SSC, 30% (v/v) formamide, 10% (w/v) dextran sulfate (Sigma‐Aldrich, cat. no. D8906), 0.1% (w/v) yeast tRNA (Thermo Fisher Scientific, cat. no. AM7119), and 1% (v/v) murine RNase inhibitor (New England BioLabs, cat. no. M0628S).

Following the overnight incubation, unbound probes were removed by washing the cells twice in the pre‐wash buffer for 30 min per wash at 37°C. For signal detection and nuclear counterstaining, the samples were incubated with 5 nM readout probes and DAPI in a secondary buffer containing 2× SSC and 10% (v/v) ethylene carbonate (Sigma‐Aldrich) for 30 min at 37°C. Finally, two additional 30‐minute washes in the pre‐wash buffer at 37°C were performed to eliminate residual background signal before imaging.

### 5.9 Western Blotting

Cells were lysed using RIPA buffer (Beyotime, cat. no. P0013B) supplemented with PMSF protease inhibitor (Beyotime, cat. no. ST505). Total protein concentrations were determined via a standard BCA assay. Equal amounts of protein lysates were resolved on 4%–20% gradient Tris‐acetate precast gels (Yeasen, cat. no. 36231ES10) at 70 V for 30 min, followed by 120 V until optimal separation was achieved. Proteins were then transferred onto 0.45 µm PVDF membranes (Millipore, cat. no. IPVH00010) via a wet transfer system (25 mM Tris, 200 mM glycine, 10% methanol) at a constant current of 200 mA for 2 h at room temperature.

Membranes were blocked with 5% non‐fat dry milk in Tris‐buffered saline containing 0.1% Tween‐20 (TBST) for 1 h at room temperature. Subsequently, membranes were incubated overnight at 4°C with primary antibodies against Flag‐tag (MCE, cat. no. HY‐P80111; 1:5,000) or β‐actin (MCE, cat. no. HY‐P80438; 1:5,000) diluted in the blocking buffer. After three 5‐minute washes in TBST, membranes were probed with an HRP‐conjugated goat anti‐mouse IgG secondary antibody (MCE, cat. no. HY‐P8004; 1:5,000) for 1 h at room temperature. Following three final TBST washes, protein bands were visualized using an ECL substrate (Thermo Scientific, cat. no. 34080) and captured with a CCD camera‐based imaging system.

### 5.10 Single‐Molecule Tracking and Imaging

For live‐cell single‐molecule imaging of RNA dynamics, cells were seeded onto 35‐mm glass‐bottom Petri dishes and cultured overnight. Prior to imaging, the culture medium was replaced with fresh phenol red‐free medium (Life Technologies) to minimize autofluorescence background. Image acquisition was performed using a Nikon Ti2‐E inverted microscope equipped with a STORM module, a 100× oil‐immersion phase‐contrast objective (UPlanSApo, NA = 1.49), and an ORCA‐Flash4.0 V3 sCMOS camera. To maintain optimal cell viability throughout the experiments, the microscope stage was enclosed in an environmental incubation chamber maintained at 37°C with a 5% CO_2_ atmosphere.

Laser power was dynamically modulated utilizing an acousto‐optic tunable filter (AOTF). To achieve high‐contrast single‐molecule visualization, highly inclined and laminated optical sheet (HILO) illumination was employed. The incident angle of the HILO beam was carefully optimized for each sample to significantly reduce out‐of‐focus cytosolic background and cellular autofluorescence. Time‐lapse image sequences were continuously acquired at an exposure time of 100 ms per frame to generate single‐molecule tracking movies.

### 5.11 Trajectory Analysis

Single‐molecule coordinate reconstruction and initial tracking were performed using the TrackMate plugin in ImageJ. The extracted molecular coordinates were subsequently analyzed using the public R package TrackmateR (https://quantixed.github.io/TrackMateR/) in RStudio.

To quantitatively evaluate imaging quality, the signal‐to‐noise ratio (SNR) of individual RNA puncta was determined. Puncta were first identified utilizing a modified Airlocalize algorithm. The SNR for each CDeg focus was then calculated using the following equation:

$$SNR=\frac{I_{peak}-I_{bg}}{\sigma_{bg}}$$

where *I_peak_* denotes the maximum peak intensity of the respective CDeg focus, while *I_bg_* and σ_*bg*_ represent the mean intensity and the standard deviation of the local cytosolic background, respectively.

The mean square displacement (MSD) of the generated trajectories was calculated using custom MATLAB scripts, as previously described. To characterize mRNA motility, the mean square displacement (MSD) for each trajectory was calculated. The nature of the motion was classified by fitting the MSD to the anomalous diffusion power law:

$$MSD\left(\tau \right)=4D\tau^{\alpha }$$

where *D* was the diffusion coefficient, τ was the time lag, and α was the anomalous diffusion scaling factor. Trajectories were categorized as sub‐diffusive (α < 1), Brownian (α  =  1), or directed (α > 1). Additionally, the fractal dimension of trajectories was calculated to quantify the space‐filling properties of mRNA movement within the crowded nuclear and cytoplasmic environments.

### 5.12 Statistical Analysis

All data were presented as means ± standard deviation (s.d.), with sample sizes (n) for each condition indicated in the respective figure legends. Statistical analyses were performed using R (RStudio). For comparisons involving more than two groups, one‐way analysis of variance (ANOVA) was applied, followed by appropriate post hoc tests: Dunnett's multiple comparison test was used when comparing multiple experimental groups against a single control group, and Tukey's honest significant difference (HSD) test was employed for all pairwise comparisons among groups. For two‐group comparisons, unpaired Student's t‐tests were used to assess significant differences between group means.

## Author Contributions

**H. Zhang** and **S. Shao** conceived and designed the experiments. **H. Zhang** performed all cloning and cell culture experiments, and **S. Shao** performed live cell imaging and single‐molecule imaging. **H. Zhang** and **S. Shao** analyzed all of the data. **H. Zhang** and **S. Shao** wrote the manuscript.

## Conflicts of Interest

The authors declare no conflicts of interest.

## Supporting information




**Supporting File 1**: advs77533‐sup‐0001‐SuppMat.docx.

**Supporting File 2**: advs77533‐sup‐0002‐Table1.xlsx.

**Supporting File 3**: advs77533‐sup‐0003‐Table2.xlsx.

**Supporting File 4**: advs77533‐sup‐0004‐Table3.xlsx.

## Data Availability

The data that support the findings of this study are available from the corresponding author upon reasonable request.
