# Supplementary material for: Single‐Molecule Imaging of Endogenous RNA Using a Miniaturized and Circularly Permuted CRISPR‐Cas13 Degron‐Masking System CDegSR
Source: Adv Sci (Weinh). 2026 Aug 31:e77533. Online ahead of print. doi: 10.1002/advs.77533 (PMC13528178; doi:10.1002/advs.77533)
Supplement: Supplementary file 1 [file ADVS-9999-e77533-s001.docx]

**Supplementary Information**

For

**Single-Molecule Imaging of Endogenous RNA Using a Miniaturized and Circularly Permuted CRISPR-Cas13 Degron-Masking System CDegSR**

*Shipeng Shao^1,2^, Hongchen Zhang ^3,#^*

*^1^ Center for Medical Epigenetics, School of Basic Medical Sciences, Children’s Hospital of Chongqing Medical University, National Clinical Research Center for Child Health and Disorder, Chongqing Medical University, Chongqing 400016, China.*

*^2^ Department of Psychiatry, Key Laboratory of Major Brain Disease and Aging Research (Ministry of Education), the First Affiliated Hospital of Chongqing Medical University, Chongqing, 400016, China.*

*^3^ Integrative Science Center of Germplasm Creation in Western China (Chongqing) Science City, MOE Key Laboratory of Freshwater Fish Reproduction and Development, School of Life Sciences, Southwest University, Chongqing 400715, China.*

^#^ Correspondence and requests for materials should be addressed to *Hongchen Zhang (Email:zhanglab2022@swu.edu.cn).*

**Supplementary Figure 1. Orthogonal Validation of dCas13-mediated *NEAT1* Labeling.**

**(a-b)** Representative fluorescence images of HeLa cells expressing d*Psp*Cas13b variants (FL, ΔHEPN1, ΔHEPN2, ΔHEPN1&2) targeting endogenous *NEAT1* lncRNA. To confirm the identity of labeled foci, cells were either immunostained for the paraspeckle marker protein PSF (a) or co-labled with FISH probe sets targeting *NEAT1* lncRNA (b). Nuclei were counterstained with DAPI. The high spatial colocalization confirms targeting specificity. Scale bars, 5 μm.

**Supplementary Figure 2. FRAP Recovery Kinetics of Engineered d*Psp*Cas13b Truncations.**

**(a–d)** Fluorescence Recovery After Photobleaching (FRAP) analysis for full-length (FL), ΔHEPN1, ΔHEPN2, and ΔHEPN1&2 variants targeting endogenous *NEAT1* transcripts. Individual paraspeckle foci were selectively photobleached to monitor protein exchange and complex stability. Individual cell trajectories are plotted alongside the ensemble average. Data represent mean ± S.D.

**Supplementary Figure 3. Incompatibility of Direct C-terminal Degron Fusion for Interaction-Dependent Stabilization.**

**(a)** Schematic illustration of the proposed mechanism for constitutive degradation in the native C-terminal configuration. In this framework, the RRRG-based Deg motif is appended directly to the native C-terminus of the miniCas13 effector.

**(b)** Overview of the experimental expression architecture used to evaluate the masking efficacy of C-terminal degron fusions. The platform utilizes a three-component system comprising the miniaturized dPspCas13b (miniCas13) scaffold, a sequence-specific crRNA, and an orthogonal Tornado-structured circular target RNA (tgRNA). This circularized target ensures stability and provides a clear benchmark for assessing target-dependent protein stabilization without interference from the endogenous transcriptome.

**(c)** Representative fluorescence images of HEK293FT cells expressing the mini-d*Psp*Cas13b-Deg fusion. To assess stabilization efficacy, fluorescence intensity were evaluated in both the absence and presence of the cognate target RNA and crRNA. The lack of signal recovery confirms that binding cannot shield the degron from intracellular degradation. Cells expressing the non-degron-tagged mini-d*Psp*Cas13b are shown as a control for baseline expression. Scale bars, 50 μm.

**(d)** Quantitative assessment of protein stability via normalized fluorescence measurement. These data confirm that simple terminal fusion is insufficient for interaction-dependent stabilization, underscoring the need for the topological rearrangement achieved by circular permutation. Significance was determined by one-way ANOVA, followed by Dunn’s multiple comparison test. For each group from left to right, n = 473, 861, 1185, and 1346 cells, respectively. ***P_mini-Deg_=2.17×10^−87^; ***P_mini-Deg+crRNA_=3.61×10^−77^; ***P_mini-Deg+crRNA+tgRNA_=1.69×10^−128^.

**Supplementary Figure 4. Comparative Analysis and Optimization of Target-Dependent Stability Across CDegSR Architectures.**

**(a)** Representative fluorescence images of HEK293FT cells expressing various CDegSR variants. To determine the efficiency of interaction-dependent degron masking, effector candidates were evaluated in both the absence and presence of the circular target RNA (Tornado-tgRNA) and its complementary crRNA. These panels illustrate the impact of linker length on basal turnover, demonstrating how fluorescence is suppressed in the unbound state and restored upon formation of the ternary ribonucleoprotein complex. Cell nuclei were counterstained with DAPI to provide morphological context. The images are representative of three independent biological replicates. Scale bars, 100 μm.

**(b)** Quantitative evaluation of variant stability through normalized fluorescence measurement. The data depict the normalized green fluorescence intensity of mBaojin-tagged CDegSR variants relative to an mCherry internal control, which was utilized to normalize for variations in transfection efficiency. Effector stability was systematically probed under three distinct conditions: the basal state (effector alone), the intermediate state (effector co-expressed with crRNA), and the functional stabilized state (effector with both crRNA and tgRNA). The statistic information is the same as in Fig. 3d.

**Supplementary Figure 5. Versatility and Modular Compatibility of the CDegSR Platform with Diverse Molecular Probes.**

**(a)** Representative fluorescence images demonstrating the fusion of various reporters with CDegSR. The CDegSR system was fused to a broad spectrum of reporters, including fluorescent proteins with different spectra (mCerulean, EGFP, and mCherry) and self-labeling enzyme tags with different dyes (HaloTag and SNAP-tag, JF549 and JF646), to assess its spectral flexibility. Each candidate was rigorously evaluated in a dual-state assay: the basal state (absence of target RNA) and the functionally stabilized state, achieved through the co-expression of a Tornado-structured circular target RNA (tgRNA) and its directing crRNA. These results illustrate that the different tags do not compromise the system's ability to undergo target-dependent signal restoration. Scale bars, 100 μm.

**(b)** Quantitative evaluation of effector stability across various protein fusions. The result confirms that the interaction-dependent degron stabilization mechanism remains highly efficient across all tested tags. This cross-compatibility establishes CDegSR as a robust, low-perturbation toolkit suitable for multicolor and multimodal RNA imaging applications. Significance was determined by two sample Wilcoxon rank sum test. For each group from left to right, n = 482, 677, 560, 391, 369, 727, 392, 486, 611, 585, 370, 669, 512, and 681 cells, respectively. ***P<2.2×10^−16^ for all group.

**Supplementary Figure 6. Robustness and Generalizability of the CDegSR Fluorogenic Switch Across Diverse Cell Lines.**

**(a, c, e)** Representative fluorescence images illustrating the target-dependent stabilization of the mini-d*Psp*Cas13b-CP423 (non-degron control) and CDegSR effectors within HeLa (a), MDA-MB-231 (c), and U2OS (e) cells. To evaluate the efficiency of interaction-dependent degron masking, fluorescence intensities were characterized in both the basal state (absence of target) and the functionally stabilized state, achieved via the co-expression of a Tornado-structured circular target RNA (tgRNA) and its cognate crRNA. Scale bars, 100 μm.

**(b, d, f)** Quantitative statistical assessment of effector stability and induction in the corresponding cell lines: HeLa (b), MDA-MB-231 (d), and U2OS (f). Fluorescence signals were normalized to internal controls to determine the fold-induction upon target engagement. These data confirm that the CDegSR maintains high-contrast signal restoration and consistent background rejection in varied cellular lines, establishing it as a versatile and robust toolkit for endogenous RNA visualization. Data are mean±S.D. from 3 independent field of views. Significance was determined by one-way ANOVA, followed by Dunn’s multiple comparison test. For each group from left to right, HeLa: n = 2047, 2457, 1595, and 1499 cells, respectively. ***P_mini-CdegSR_<2.2×10^−16^; ***P_mini-CDegSR+crRNA_<2.2×10^−16^; n.s. P_mini-CDegSR+crRNA+tgRNA_=0.51. MDA-MB-231: n = 1879, 2276, 1434, and 1156 cells, respectively. ***P_mini-CdegSR_<2.2×10^−16^; ***P_mini-CDegSR+crRNA_<2.2×10^−16^; n.s. P_mini-CDegSR+crRNA+tgRNA_=0.29. U2OS: n = 2150, 2456, 1779, and 1990 cells, respectively. ***P_mini-CdegSR_<2.2×10^−16^; ***P_mini-CDegSR+crRNA_<2.2×10^−16^; n.s. P_mini-CDegSR+crRNA+tgRNA_=1.17×10^−5^.

**Supplementary Figure 7. *NEAT1* labeling using CDegSR across distinct cell lines.**

Representative fluorescence images of 293FT, MDA-MB-231, and U2OS cells expressing CDegSR targeting endogenous *NEAT1* lncRNA.Cells were also co-labled with FISH probe sets targeting *NEAT1* lncRNA. Nuclei were counterstained with DAPI. Scale bars, 5 μm.

**Supplementary Figure 8. Strategy for the Sequential Multimerization of ALFA-tag Units for CDegSR Imaging.**

**(a)** Schematic representation of restriction cleavage and ligation. The diagram illustrates the compatible cohesive ends generated by the isocaudomers *Bgl*II and *BamH*I. Fusion of these complementary overhangs creates a novel hybrid junction that is no longer recognized or cleaved by either of the original restriction endonucleases.

**(b)** Workflow of the directional cloning approach. This panel outlines the step-by-step assembly of tandem ALFA-tags. Each building block features an ALFA-tag coding sequence (SRLEEELRRRLTE) paired with a flexible linker, which is specifically designed to prevent spatial interference among the recruited CDeg effectors. During each multimerization cycle, the recipient vector harboring the existing repeat(s) is linearized via *BamH*I and *EcoR*I digestion. Concurrently, the incoming sequence is excised using both *Bgl*II and *EcoR*I. Ligation of these compatible ends results in an uncleavable scar. Crucially, the intact *Bgl*II and *BamH*I recognition sites are maintained exclusively at the 5' and 3' ends of the newly formed array, thereby permitting unlimited rounds of iterative insertion.

**(c)** Agarose gel electrophoresis of the engineered constructs. The successful assembly of plasmids carrying varying numbers of ALFA repeats is confirmed via restriction mapping. To verify the insert sizes, the final plasmids were subjected to double digestion with *Bgl*II and *BamH*I, effectively releasing the entire multimerized cassette. The GoldBand DL10,000 DNA Marker (Yeasen, cat. no. 10505ES60) was included as a molecular weight reference.

**Figure 9. Parallel Comparison of CDegSR and MS2-mediated Platforms for Single-molecule Tracking of Identical Transcripts.**

**(a, b)** Schematic illustration of the comparative labeling strategies utilizing the CDegSR platform and the gold-standard MS2 system on an identical mRNA species. The coding sequence of POLR2A was appended with a 24× MS2 cassette in its 3′ UTR, allowing the same reporter transcript to be independently tracked via either tdMCP-GFP (a) or tiled CDegSR effectors (b). **(c, d)** Representative live-cell trajectories of individual POLR2A-24×MS2 mRNA molecules resolved by tdMCP-GFP (c) and CDegSR (d). Trajectories are color-coded according to the magnitude of their local diffusion coefficients. **(e–h)** Comprehensive statistical analysis of mRNA diffusion kinetics using 4 biophysical parameters: jumping distance (e) ,diffusion coefficient (f) , anomalous diffusion exponent (g), and fractal dimension (h). Statistical significance was determined using a two-sample Wilcoxon rank-sum test. Box-and-hist plots represent data distributions from n = 6145 tracked molecules for the MS2 system and n = 5471 tracked molecules for the CDegSR system collected from independent experiments.
